# 

**Published:** 1895-11

**Authors:** 


					﻿THE
Southern Medical Record.
A Monthly Journal of Medicine and Surgery.
Vol. XXV. ATLANTA, GA., NOVEMBER, 1895.	No. 11?
BERNARD WOLFF, M. D., Editor.
LOUIS H. JONES, M; D., Business Manager.
Associate Editors :
A W. GRIGGS, M. D. • -	WILLIS F. WESTMORELAND, M. D.
J. McFADDEN GASTON, M. D.
Entered at the Post-office at Atlanta, G^., as Second-Class Matter.
The Foote & Davies Co., Atlanta, Ga.
				

